# Factors affecting emerging infectious disease-related infection control performance of nurses working in national and public hospitals: a cross-sectional survey

**DOI:** 10.1186/s12912-026-04814-6

**Published:** 2026-05-28

**Authors:** Jae Eun Ha, Ju Young Park

**Affiliations:** 1 Busan Veterans Hospital, Busan, South Korea; 2 College of Nursing, Konyang University, Daejeon, South Korea

**Keywords:** Emerging infectious diseases, Infection control, Nurses, Moral sensitivity, Burnout, Infection prevention environment, Public hospitals, Cross-sectional study

## Abstract

**Background:**

Emerging infectious diseases pose serious threats to global health, and nurses working in national and public hospitals play a pivotal role in infection prevention. However, their infection control performance can be influenced by various psychosocial and environmental factors.

**Methods:**

This cross-sectional study surveyed 193 nurses from three national and three public hospitals in South Korea. Data were collected in May 2022 using validated self-report questionnaires measuring burnout, moral sensitivity, infection prevention environment, and infection control performance related to emerging infectious diseases. Statistical analyses included descriptive statistics, Pearson’s correlation, and hierarchical regression.

**Results:**

The mean infection control performance score was 4.37 (SD = 0.53) out of 5. Moral sensitivity (β = 0.16, *p* = .014) and infection prevention environment (β = 0.48, *p* < .001) significantly predicted infection control performance, explaining 37% of the variance. Burnout showed a negative correlation but was not a significant predictor in the regression model.

**Conclusions:**

Infection control performance among nurses in national and public hospitals is significantly affected by moral sensitivity and the institutional infection prevention environment. Interventions to enhance these factors are critical to improving infection control during emerging infectious disease outbreaks.

**Clinical trial number:**

Not applicable.

## Introduction

Emerging infectious diseases (EIDs), such as COVID-19 and MERS, pose complex challenges to healthcare systems, requiring not only rapid response but also consistent adherence to fundamental infection control practices. During the COVID-19 pandemic, healthcare workers experienced a substantial risk of infection, highlighting the importance of effective infection control measures [1]. Nurses are particularly vulnerable due to prolonged and close contact with infected patients, making their role in infection prevention critical. Although EIDs present novel clinical and organizational challenges, their management largely depends on the rigorous implementation of established infection prevention principles, including isolation precautions, appropriate use of personal protective equipment, and environmental control. Previous studies have shown that inadequate adherence to infection control guidelines, particularly during the use of personal protective equipment, increases the risk of transmission [2].

In South Korea, national and public hospitals play a central role in responding to public health emergencies, as they are officially designated to manage patients with emerging infectious diseases. Nurses working in these institutions are more likely to engage in high-intensity infection control activities, often under resource constraints and ethical pressures. Despite their critical role, relatively limited research has focused specifically on infection control performance within this public healthcare context. Previous studies have identified various psychosocial and organizational factors influencing infection control performance among nurses. However, most prior research has focused on general clinical settings, with limited attention to these factors in the context of emerging infectious diseases. Furthermore, these findings have not been sufficiently integrated within the broader international literature on infection control and healthcare system preparedness.

Therefore, this study aims to examine the relationships among burnout, moral sensitivity, infection prevention environment, and infection control performance among nurses working in national and public hospitals, in order to provide evidence for improving infection control practices in the context of emerging infectious diseases.

## Background

Nurses in nationally designated infectious disease hospitals spend 51.6% of their direct nursing time on donning and doffing Level D personal protective equipment, as well as disinfection and waste management which raises concerns about burnout [3]. Such prolonged and repetitive infection control activities may contribute to physical and emotional exhaustion. Previous studies have indicated that burnout is associated with reduced infection control performance among general nurses [4]. Nurses experiencing high levels of burnout may be less likely to detect potential risks early and may demonstrate decreased professional competency, thereby compromising patient safety activities related to infection control [5]. Moreover, reductions in nurse burnout have been associated with improvements in infection control performance and decreases in infection rates and healthcare costs [6]. These findings suggest that burnout may be an important factor associated with infection control performance, particularly in high-intensity infectious disease settings.

Moral sensitivity refers to nurses’ ability to discern moral problems in clinical situations and to recognize and respond to ethical conflicts [7]. It involves recognizing ethical issues embedded in clinical practice and understanding how one’s actions may affect patients [8, 9]. Nurses with high moral sensitivity are better able to make responsible decisions when faced with ethical dilemmas, whereas those with low sensitivity may fail to recognize ethically significant aspects of care [9]. While moral sensitivity is a prerequisite for ethical competence, contextual and organizational factors may weaken moral sensitivity, thereby negatively affecting the quality of care provided [10]. According to Lützén’s ethical decision-making framework, moral sensitivity represents an essential first step in ethical competence because it enables healthcare professionals to identify moral conflicts prior to decision-making [8]. Without adequate moral sensitivity, nurses may fail to perceive ethically significant aspects of infection control situations, particularly in high-risk and resource-constrained environments.

Previous studies have reported that moral sensitivity is associated with compliance with standard precautions and infection control behaviors [11]. Research conducted in small and medium hospitals demonstrated a positive correlation between moral sensitivity and adherence to fundamental infection control standards [12], while studies in long-term care hospitals identified moral sensitivity as a significant predictor of infection control performance [13]. Furthermore, in COVID-19 wards, moral sensitivity showed a strong association with overall nursing performance [14]. However, most prior studies have been conducted in general hospital settings or focused on standard precautions rather than infection control performance specifically related to emerging infectious diseases. Therefore, further investigation is warranted to examine the association between moral sensitivity and infection control performance in nationally designated infectious disease hospitals.

Infection control during emerging infectious disease outbreaks involves ethical challenges that extend beyond technical guideline adherence. Nurses may encounter dilemmas related to balancing patient autonomy with isolation measures, allocating limited PPE resources, reconciling professional duty with self-protection, and managing moral distress during high-risk procedures. Additionally, maintaining patient confidentiality while ensuring timely disclosure of infection status presents further ethical complexity. These ethical tensions indicate that infection control performance in emerging infectious disease contexts requires not only technical competence but also ethical judgment.

In clinical practice, adherence to infection control guidelines does not always correspond to expected standards, suggesting the presence of a knowledge–practice gap. Psychological and ethical factors may influence compliance beyond mere knowledge of guidelines [11]. Moral sensitivity may therefore facilitate the translation of infection control knowledge into practice, particularly in ethically complex situations.

Furthermore, healthcare institutions provide spatial environments for patient treatment. However, these environments can also pose risks for transmitting infections to potentially immunocompromised patients, elderly patients, and healthcare workers [15]. The infection prevention environment encompasses facilities, equipment, and administrative support that enable healthcare workers to protect themselves from infectious exposure during care and testing processes [16]. Well-established infection prevention environments may enhance trust in the workplace, reduce anxiety related to infection risks, and promote compliance with preventive practices [17]. Prior research indicates that turnover intention is higher in settings with low infection prevention environments [17], while compliance with standard practices improves in environments with stronger infection prevention support [18]. However, limited evidence exists regarding how the infection prevention environment is associated with infection control performance specifically in the context of emerging infectious diseases in nationally designated hospitals.

In this study, we aimed to examine the associations between burnout, moral sensitivity, the infection prevention environment, and infection control performance related to emerging infectious diseases among nurses working in national and public hospitals designated for infectious diseases. This research seeks to provide evidence to inform strategies for strengthening preventive responses to emerging and re-emerging infectious diseases.

## Methods

### Design and participants

This study employed a cross-sectional descriptive design to identify predictors of infection control performance among nurses working in national and public hospitals in South Korea. A total of 193 nurses who were directly involved in patient care were recruited through convenience sampling. Eligibility criteria included having at least six months of clinical experience and providing informed consent to participate in the study.

The required sample size was calculated using G*Power 3.1.2 for multiple regression analysis. Based on a previous study [19], an effect size of 0.15, a significance level of 0.05, and a power of 0.95 were applied, resulting in a minimum sample size of 183. Considering a 10% potential dropout rate, 211 participants were initially recruited. After excluding incomplete responses, data from 193 participants were included in the final analysis.

### Instruments

#### Infection control performance related to emerging infectious diseases

In this study, infection control performance related to emerging infectious diseases refers to the degree to which nurses adhere to core infection prevention and control practices—such as isolation precautions, use of personal protective equipment, and transmission-based precautions. These practices constitute fundamental components of infection control applicable to both general and emerging infectious disease situations and are applied in this study as operational responses within the context of emerging infectious diseases.

The infection control performance related to emerging infectious diseases utilized a modified and supplemented tool based on the MERS isolation guidelines developed by Kim and Song [20]. This tool was approved by Yi and Cha [19] for assessing infection control guideline adherence for emerging infectious diseases among emergency room nurses.

The tool consists of a total of 18 items covering screening, isolation and reporting, donning and doffing personal protective equipment (PPE), specimen collection and management, aerosol-generating procedures, management of family members and visitors, patient transport and movement, environmental management, and medical waste disposal.

Conceptually, these items reflect core domains of infection prevention and control, including [1] early detection and reporting [2], implementation of isolation and transmission-based precautions [3], appropriate use of personal protective equipment [4], management of high-risk clinical procedures, and [5] environmental and waste control. These domains correspond to the fundamental components of national and international infection control guidelines, such as standard precautions and transmission-based precautions, which are essential for preventing the spread of emerging infectious diseases in healthcare settings. Therefore, this tool was considered appropriate for operationalizing infection control performance in nationally designated infectious disease hospitals.

Responses are measured on a 5-point Likert scale, ranging from 5 points for “Always perform” to 1 point for “Never perform,” indicating that a higher score reflects a higher level of infection control performance related to emerging infectious diseases. The Cronbach’s alpha reported by Kim and Song [20] was 0.93, while that in the study by Yi and Cha [19] was 0.90. In this study, the Cronbach’s alpha was 0.92.

#### Burnout

Burnout was measured using a tool that was modified and supplemented by Moon and Han [21] based on the original instrument developed by Pines, Aronson, and Kafry [22]. This tool consists of a total of 20 items divided into three subdomains: physical exhaustion with 6 items, emotional exhaustion with 7 items, and mental exhaustion with 7 items. Responses are measured on a 5-point Likert scale, ranging from 5 points for “Strongly agree” to 1 point for “Not at all,” with positive items being reverse-coded. A higher score indicates a higher level of burnout. The reliability of the tool was reported as a Cronbach’s alpha of 0.91 at the time of development, 0.85 in the study by Moon and Han [21], and 0.85 in the current study.

#### Moral sensitivity

Moral sensitivity was defined as the ability to recognize moral problems, intuitively understand patients’ vulnerable situations, and interpret clinical situations in ways that support ethically appropriate decision-making [8]. In this study, moral sensitivity was operationally defined as the score measured using the Korean version of the Moral Sensitivity Questionnaire (K-MSQ). We utilized the Moral Sensitivity Questionnaire (K-MSQ), which was translated, adapted, and validated by Han et al. [23] based on the original instrument developed by Lutzen et al. [8]. The K-MSQ consists of 27 items categorized into five subdomains: patient-centered care (5 items), professional responsibility (7 items), conflict (5 items), moral significance (5 items), and benevolence (5 items). These domains reflect key dimensions of moral sensitivity in clinical practice, including awareness of patients’ needs, professional accountability in ethical decision-making, recognition of moral conflicts, understanding of moral values in care situations, and commitment to acting in the patient’s best interests. Responses are measured on a 7-point Likert scale ranging from 7 (“strongly agree”) to 1 (“strongly disagree”), with higher scores indicating higher levels of moral sensitivity. In Han et al.‘s study [23], Cronbach’s alpha was 0.76, while in the present study it was 0.86.

#### Infection prevention environment

We measured the infection prevention environment related to emerging infectious diseases using a defense environment measurement tool developed by Han [24]. This tool was modified and supplemented by Ahn, Kim, & Kim [16] to address the issue of defense environments concerning infection exposure among emergency room nurses, and approval for its use was obtained. The tool consists of a total of 11 items, which include the availability of personal protective equipment, the interest of healthcare facility managers in infection exposure, and elements related to health check-ups and vaccinations. Each item is measured on a 5-point Likert scale, ranging from 1 point for “Not at all” to 5 points for “Very much,” with higher scores indicating a better infection exposure prevention environment. In Han’s study [24], the reliability was reported with a Cronbach’s alpha of 0.89, while Ahn et al. [16] reported a Cronbach’s alpha of 0.85. In the current study, the Cronbach’s alpha was 0.89.

### Data collection and ethical considerations

This cross-sectional descriptive study was conducted after obtaining research approval from the Institutional Review Board of REDACTED in D City. The data collection period was from May 11, 2022, the date of IRB approval, to May 19, 2022. For recruitment, the researcher explained the purpose and content of the study to the heads of the nursing departments at P Veterans Hospital and P Medical Center located in Pusan City, obtained their approval, and then commenced data collection. The researcher informed the participants that they could voluntarily withdraw from participation at any time without facing any disadvantages, and obtained their consent. Questionnaires were distributed in quantities of 15 to 25 per ward, and upon completion, the questionnaires were sealed in individual envelopes and stored in each department until collected by the researcher. Participants in the study received a small token of appreciation.

### Data analysis

The SPSS Statistics 28.0 software was used for the analyses. Frequency, percentage, mean, and standard deviation were used to assess the general characteristics of the participants, as well as their infection control performance related to emerging infectious diseases, burnout, moral sensitivity, and infection prevention environment. Differences in the level of infection control performance related to emerging infectious diseases according to the general characteristics of the participants were analyzed using the Independent t-test, One-way ANOVA, and Scheffé post-hoc test. The correlation between burnout, moral sensitivity, infection prevention environment, and infection control performance related to emerging infectious diseases was analyzed using Pearson’s Correlation Coefficient. Hierarchical regression analysis was utilized to identify the factors affecting the infection control performance related to emerging infectious diseases among the participants. The level of significance was set at 0.05 for all statistical tests.

## RESULTS

### General characteristics of participants

The general characteristics of the participants are presented in Table 1. Participants were predominantly female staff nurses working in general wards of general hospitals. Most held a bachelor’s degree, and clinical experience varied widely among participants. A large proportion reported experience caring for patients with emerging infectious diseases and having received education related to infection control guidelines. Detailed distributions are shown in Table 1.

**Table 1:** Participants’ general characteristics (*N* = 193)

Characteristics	Categories	*n* (%)	Mean ± SD
	≤ 25	35(18.2)	
Age (yr)	26∼30	74(38.3)	31.92 ± 7.31
	31∼35	34(17.6)	
	3 ≥ 36	50(25.9)	
Gender	Female	180(93.3)	
	Male	13(6.7)	
	Diploma	30(15.5)	
Education level	Bachelor	158(81.9)	
	Master’s degree	5(2.6)	
Marital status	Single	118(61.1)	
	Married	75(38.9)	
	General nurse	171(88.6)	
Position	Chief nurse	14(7.3)	
	Head nurse	8(4.1)	
Type of medical institution	General hospital	193(100.0%)	
	< 3	59(30.6)	
Clinical career (yr)	3 to < 6	47(24.3)	7.88 ± 7.58
	6 to < 10	27(14)	
	≥ 10	60(31.1)	
Working experience of	< 1	29(15.0)	
current work	1 to < 2	60(31.1)	2.56 ± 2.47
	2 to < 3	32(16.6)	
place (yr)	≥ 3	72(37.3)	
	Isolation ward	64(33.2)	
Work unit	Emergency room	12(6.2)	
	General ward	111(57.5)	
	Others	6(3.1)	
Screening station	Yes	184(95.3)	
	No	9(4.7)	
General isolation room (not emerging infectious disease)	Yes	175(90.7)	
	No	18(9.3)	
Experience with	Yes	182(94.3)	
emerging infectious disease patients care	No	11(5.7)	
Experience with infection of emerging infectious disease	Yes	119(61.7)	
	No	74(38.3)	
Monitoring and feedback	Yes	172(89.1)	
Personal protect equipment	No	21(10.9)	
Education completion	Yes	182(94.3)	
Related to emerging infectious disease	No	11(5.7)	

### Levels of burnout, moral sensitivity, infection prevention environment, and infection control performance

The levels of burnout, moral sensitivity, infection prevention environment, and infection control performance related to emerging infectious diseases are summarized in Table 2. Overall, participants reported moderate levels of burnout and relatively high levels of moral sensitivity, infection prevention environment, and infection control performance.

**Table 2:** Descriptive statistics of the variables (*N* = 193)

Variables	Mean ± SD	Min	Max	Range
Burnout	3.12 ± 0.47	2	4	1∼5
Physical exhaustion	3.73 ± 0.62	2	5	
Emotional exhaustion	2.68 ± 0.57	1	5	
Mental exhaustion	3.04 ± 0.50	2	4	
Moral sensitivity	4.92 ± 0.55	4	7	1∼7
Patient centered nursing	5.30 ± 0.74	4	7	
Professional responsibility	5.28 ± 0.67	3	7	
Conflict	4.72 ± 0.79	3	7	
Moral meaning	4.26 ± 0.92	2	7	
Beneficence	4.92 ± 0.63	3	7	
Infection prevention environment	4.13 ± 0.56	2	5	1∼5
Personal protective equipment	4.15 ± 0.63	2	5	
Organizational support	4.03 ± 0.88	1	5	
Disinfection and sterilization	3.75 ± 1.06	1	5	
Education of infection control	3.97 ± 0.82	1	5	
Instruction protocol	4.10 ± 0.77	1	5	
Protocol	4.01 ± 0.83	2	5	
Damaged waste management	4.58 ± 0.66	1	5	
Health screenings	4.40 ± 0.71	2	5	
Vaccination	4.18 ± 0.83	2	5	
Performance of infection control related to emerging infectious diseases	4.37 ± 0.53	3	5	1∼5
Screening	3.87 ± 0.87	1	5	
Isolation and Report	4.12 ± 0.90	1	5	
Duffing and donning off personal protective equipment	4.53 ± 0.55	2	5	
Procedures that generate aerosols	4.27 ± 0.81	2	5	
Sample collection and management	4.47 ± 0.61	3	5	
Family and Visitor infection control	4.48 ± 0.63	3	5	
Patient transport and movement	4.51 ± 0.66	1	5	
Environmental infection control	4.53 ± 0.60	3	5	
Disposal of contaminated materials	4.56 ± 0.59	2	5	

### Differences in infection control performance according to general characteristics

Significant differences in infection control performance related to emerging infectious diseases were found according to age, position, and monitoring and feedback during donning and doffing of personal protective equipment. Older nurses and those in higher positions demonstrated higher levels of infection control performance. In addition, participants who received monitoring and feedback during personal protective equipment use showed significantly higher performance compared with those who did not (Table 3).

**Table 3:** Differences in performance of infection control related to emerging infectious diseases according to the subject’s general characteristics (*N* = 193)

Variables	Categories	Performance of infection control related to emerging infectious diseases				
		Mean ± SD	Median	Min	Max	t or F(*p*) Scheffé
Gender	Female	4.38 ± 0.50	4.50	3.0	5.0	0.77
	Male	4.26 ± 0.67	4.44	3.0	5.0	(0.113)
Age (yr)						3.75 (0.012) b < d
	≤ 25^a^	4.42 ± 0.47	4.50	3.1	5.0	
	26∼30^b^	4.24 ± 0.55	4.19	3.0	5.0	
	31∼35^c^	4.35 ± 0.51	4.44	3.0	5.0	
	≥ 36^d^	4.54 ± 0.42	4.61	3.5	5.0	
	Diploma	4.39 ± 0.55	4.50	3.1	5.0	0.19 (0.828)
Education level	Bachelor	4.36 ± 0.50	4.50	3.0	5.0	
	Master’s Degree	4.50 ± 0.43	4.61	3.8	5.0	
Marital status	Single	4.33 ± 0.52	4.42	3.0	5.0	-1.42
	Married	4.44 ± 0.49	4.50	3.0	5.0	(0.156)
	General nurse^a^	4.32 ± 0.51	4.39	3.0	5.0	7.89
Position	Chief nurse^b^	4.71 ± 0.32	4.83	3.9	5.0	(< 0.001)
	Head nurse^c^	4.85 ± 0.18	4.92	4.5	5.0	a < b,c

### Correlations among study variables

Correlation analysis revealed that infection control performance related to emerging infectious diseases was negatively associated with burnout and positively associated with moral sensitivity and infection prevention environment. Among these variables, infection prevention environment showed the strongest positive correlation with infection control performance (Table 4).

**Table 4:** Pearson’s correlation analysis among the variables (*N* = 193)

Variables	Burnout	Moral sensitivity	Infection prevention environment	Performance of infection control related to emerging infectious diseases
	*r*(*p*)			
Burnout	1			
Moral sensitivity	− 0.20 (0.004)	1		
Infection prevention environment	− 0.35 (< 0.001)	0.29 (< 0.001)	1	
Performance of infection control related to emerging infectious diseases	− 0.30 (< 0.001)	0.33 (< 0.001)	0.59 (< 0.001)	1

### Factors influencing infection control performance

Hierarchical regression analysis was conducted to identify factors influencing infection control performance related to emerging infectious diseases. After controlling for significant general characteristics, infection prevention environment and moral sensitivity remained significant predictors. Infection prevention environment showed the strongest effect, followed by moral sensitivity. The final model explained 37% of the variance in infection control performance (Table 5).

**Table 5:** Factors affecting performance of infection control related to emerging infectious diseases (*N* = 193)

Variables	Model 1						Model 2					
	B	SE	β		t	*p*	B	SE	β		t	*p*
(constant)	4.07	0.24			17.27	< 0.001	1.95	0.48			4.06	< 0.001
Age	0.00	0.01	0.00		0.01	0.992	0.00	0.00	− 0.00		− 0.04	0.968
Clinical career												
Chief nurse	0.35	0.18	0.18		1.99	0.049	0.06	0.15	0.03		0.40	0.689
Head nurse	0.49	0.22	0.19		2.25	0.025	0.12	0.19	0.05		0.63	0.527
Monitoring and feedback of Personal protective equipment	0.29	0.11	0.17		2.51	0.013	0.17	0.09	0.11		1.79	0.075
Burnout							− 0.08	0.06	− 0.08		-1.22	0.226
Moral sensitivity							0.15	0.06	0.16		2.49	0.014
Infection prevention environment							0.44	0.06	0.48		7.29	< 0.001
F(*p)*	5.61(< 0.001)						16.83(< 0.001)					
R^2^	0.11(0.09)						0.39					
Adj R^2^	0.09						0.37					
Multicollinearity	Durbin-Watson			1.83								
	VIF			1.05–2.19								

† Dummy variable: Position (General nurse = 0, Chief nurse, Head nurse = 1), Monitoring and feedback of personal protective equipment (No = 0, Yes = 1)

## Discussion

This study aimed to confirm the infection control performance related to emerging infectious diseases among nurses in national and public hospitals, identify the effects of burnout, moral sensitivity, and infection prevention environment on this performance, and seek measures to improve infection control performance. The major findings of this study are discussed under the following themes: infection control performance levels, key psychosocial and environmental factors, and implications for improving infection control practice.

### Infection control performance related to emerging infectious diseases

The infection control performance related to emerging infectious diseases among the participants in this study was measured at 4.37 out of a maximum of 5 points. This result is similar to the 4.33 points reported in a study by Yi and Cha [19], which used the same tool for emergency room nurses. Although the tools were not identical, a comparison with the MERS isolation guideline performance of general nurses [20] showed a lower average score. These findings suggest that infection control performance among nurses in national and public hospitals is relatively high, possibly reflecting increased awareness and preparedness following recent emerging infectious disease outbreaks.

However, it should be considered that since this study used self-reported questionnaires instead of direct observations, the actual infection control performance may differ from the reported results. Previous studies have shown discrepancies between self-reported compliance and observed compliance. For example, Kim [25] reported self-reported compliance at 95%, whereas observed compliance was 76.2%. Therefore, future studies should incorporate direct observational methods to obtain more objective assessments of infection control performance.

In this study, the lowest performing subdomain of infection control related to emerging infectious diseases was screening and isolation and reporting. In contrast, previous research [19] found that these domains were rated highly among emergency room nurses. This difference may reflect variations in clinical roles and responsibilities across departments. Emergency room nurses are required to perform triage using the Korean Triage and Acuity Scale (KTAS) [26], which mandates screening and isolation before treatment. Meanwhile, medical waste disposal and donning and doffing personal protective equipment showed the highest performance. This may reflect repeated education, institutional emphasis, and improved accessibility to infection control resources following the MERS outbreak and the COVID-19 pandemic.

### Burnout among nurses caring for emerging infectious disease patients

Participants’ burnout was measured at 3.12 out of 5 points, representing above-average levels. This is higher than the 2.96 points found in a study by Eo et al. [27] using the same tool, and while direct comparison is difficult as different tools were used, a study of nurses in designated COVID-19 treatment beds [28] found burnout at 2.71 points out of 6 (which translates to 2.26 out of 5), and another study measuring burnout in university hospital nurses [29] found it at 2.97 points out of 5. Such results may reflect the fact that the data collection for this study occurred 2 years after the declaration of the COVID-19 pandemic, during which nurses were involved in caring for suspected or confirmed patients, thereby impacting these results. Proper training for caring for infectious disease patients influences the burnout of healthcare workers [30], indicating a need to develop support systems and training programs that minimize burnout in caring for patients with emerging infectious diseases. In this study, physical exhaustion among subdomains of burnout was reported as the highest, which can be attributed to wearing unfamiliar personal protective equipment and undertaking more intensive and complex tasks compared to typical patient care when managing suspected or confirmed infectious disease patients. Therefore, to maintain an appropriate nurse-to-patient ratio in infectious disease units, it is essential to secure nursing personnel, and government policies, such as differential nursing management fees, should be prioritized.

### Moral sensitivity in infection control practice

Moral sensitivity was measured at 4.92 out of a maximum of 7 points, indicating above-average levels. This finding is consistent with a previous study conducted among general nurses by Eom and Kim [32], which reported a similar level of moral sensitivity (4.96). In addition, prior research has demonstrated that moral sensitivity is a significant factor influencing nurses’ safety-related behaviors and professional outcomes [31]. Therefore, the level of moral sensitivity observed in this study suggests that it may contribute positively to nurses’ engagement in safety-related practices. However, this study’s result is slightly lower than the 5.05 reported in the research by Han et al. [11] involving hospital nurses. In line with multiple prior studies [11, 31, 32], the patient-centered care domain showed the highest score among the dimensions of moral sensitivity in this study. In contrast, the moral significance dimension had the lowest score. This can be interpreted as the nurses primarily considering patients’ autonomy when making ethical decisions during their duties, thus leading to high scores in patient-centered care. However, while frequently exposed to situations involving moral dilemmas while caring for confirmed or suspected patients with emerging infectious diseases, their sensitivity may decrease, resulting in lower measurements of moral significance. Based on this, it is necessary to emphasize the moral significance aspect in intervention strategies to enhance the moral sensitivity of nurses in national and public hospitals.

### Infection prevention environment and institutional support

The infection prevention environment scored 4.13 out of a maximum of 5 points. In similar studies using the same tool, emergency room nurses had an average score of 3.99 [16], while hospital nurses showed a score of 4.0 [33], indicating a generally higher assessment. This increase may be attributed to the heightened awareness of the risks of infectious disease transmission due to the 2015 MERS outbreak and the ongoing COVID-19 pandemic, leading to an emphasis on the need for systematic infection management guidelines along with sufficient resources and equipment and the expansion of negative pressure facilities [34]. The highest scoring item in the subdomains of the infection prevention environment in this study was hazardous waste management, which is consistent with findings from a 2018 study on exposure to infection-related behaviors [16]. The improvement in performance related to hazardous waste management is likely due to making dedicated containers available for the easy separation and collection of sharp objects, as well as familiarity with systematic protocols in cases of needlestick injuries and repeated training. The lowest scoring item in the subdomain of the infection prevention environment was the lack of equipment for disinfection and sterilization. While the importance of environmental improvements and infection management within healthcare institutions has been emphasized due to the repeated emergence of infectious diseases, it is evident that accessibility to disinfectants and sterilization equipment is low in actual practice. Therefore, to establish a systematic infection management system in healthcare institutions, it is necessary to ensure the availability of disinfection and sterilization supplies and to renovate facilities for better accessibility.

When examining the differences in infection control performance related to emerging infectious diseases according to the participants’ general characteristics, those aged 36 years and older showed higher performance compared to those aged 26 to 30. Similarly, charge nurses and head/responsible nurses showed higher performance than staff nurses. This finding aligns with a study on the use of face shields by nurses [35], which indicated that clinical adherence to personal protective equipment increases by 2% for every additional year of clinical experience. Additionally, in a study on respiratory infections involving general nurses [36], it was found that higher age and increased clinical experience significantly enhanced adherence to personal protective equipment. Moreover, nurses who received education related to infection management had higher infection control performance than those who did not [20, 35], and those who received monitoring and feedback during the donning and doffing of personal protective equipment exhibited better performance than those who did not [19].

These results indicate the necessity to strengthen education related to infection control performance among nurses with lower age and position, particularly among those who do not receive monitoring and feedback regarding donning and doffing personal protective equipment. There is a need to develop educational programs while considering general characteristics. Most importantly, monitoring and feedback regarding donning and doffing personal protective equipment should not be a one-time event during emerging disease outbreaks but should be systematic and conducted regularly to prepare for future occurrences of new or mutated infectious diseases.

### Factors influencing infection control performance

In this study, the factors affecting infection control performance related to emerging infectious diseases were the infection prevention environment and moral sensitivity. This finding is consistent with previous research [14, 37], which indicated that the infection prevention environment influences the improvement of infection management performance. However, in some earlier studies [11, 12, 38], while moral sensitivity was correlated with standard infection control practices, it was not identified as a significant influencing factor on standard infection management performance. Moral factors can yield various outcomes because they are complex and multifactorial [10]; thus, it is necessary to verify the impact of moral sensitivity on infection control performance related to emerging infectious diseases in clinical situations similar to this study. On the other hand, participants’ burnout was found to have a negative correlation with infection control performance related to emerging infectious diseases, but it was not a significant influencing factor in the hierarchical regression model. This is consistent with the findings of Yi and Cha [19]. Although burnout was negatively correlated with infection control performance, it was not a significant predictor in the hierarchical regression model. This discrepancy may be explained by the influence of organizational factors, particularly the infection prevention environment. When such contextual variables are included in the model, their stronger explanatory power may attenuate or suppress the effect of individual-level factors such as burnout. This finding suggests that infection control performance is shaped not only by individual psychological factors but also by organizational conditions, highlighting the importance of a supportive infection prevention environment.

However, these findings differ from those of a study on Ecuadorian nurses [4], in which burnout was identified as a negative influencing factor on infection control performance, suggesting that further research is needed to clarify these relationships.

Overall, our findings suggest that to enhance the infection control performance related to emerging infectious diseases among nurses in national and public hospitals, efforts should be focused on increasing moral sensitivity rather than solely concentrating on addressing burnout. Therefore, it is essential to develop systematic educational programs aimed at fostering moral sensitivity among nurses in national and public hospitals, as well as securing administrative support from healthcare institutions to establish an infection prevention environment that allows for safe care of patients with emerging infectious diseases.

These findings should be interpreted within the broader global context of infection control in healthcare systems. International studies have highlighted that nurses’ infection control performance is influenced not only by individual factors but also by organizational conditions such as workload, staffing levels, and institutional support [6, 39]. In particular, public healthcare settings often face structural challenges, including resource constraints and increased responsibility for infectious disease response, which may affect infection prevention practices. In line with this perspective, our findings suggest that infection prevention environment and institutional support play a critical role in facilitating effective infection control performance among nurses [6, 39]. These results indicate that improving infection control performance requires not only enhancing individual competencies but also strengthening organizational systems and policy-level support. Therefore, targeted interventions at both the institutional and policy levels are necessary to ensure sustainable and effective infection control practices, particularly in public healthcare settings.

### Study limitations

This study has several limitations. First, as the participants were recruited from six national and public hospitals, caution is needed when generalizing the findings. Second, because this study employed a cross-sectional design, causal relationships between variables cannot be established. Third, infection control performance was measured using self-reported questionnaires rather than direct observation, which may have introduced response bias. In addition, the mean infection control performance score was relatively high, suggesting a potential ceiling effect. This may be partly attributable to social desirability bias inherent in self-reported measures, as participants may have overreported their adherence to infection control practices. Furthermore, the use of self-reported data rather than direct observational methods may have led to an overestimation of actual performance. Additionally, the data collection period was relatively short (May 11–19, 2022), which may limit the ability to capture stable patterns in the variables of interest and affect the generalizability of the findings. The results may reflect specific conditions during the data collection period rather than broader trends and may have been influenced by temporary situational factors. Therefore, future studies should incorporate objective measures, such as direct observation or audit-based assessments, and consider longer data collection periods to provide a more accurate evaluation of infection control performance.

## Conclusion

According to the findings of our study, the infection prevention environment and moral sensitivity were identified as significant predictors of nurses’ infection control performance related to emerging infectious diseases in national and public hospitals. There is a need to implement customized programs aimed at enhancing nurses’ ongoing moral sensitivity through effective monitoring and feedback on personal protective equipment (PPE) usage and donning practices. To improve infection control outcomes related to emerging infectious diseases, the enhancement of the infection prevention environment must be addressed, specifically tailored to job characteristics and supported by institutional administration. Our study emphasizes that addressing these critical issues is essential for fostering effective infection control practices among nurses.

In addition, we suggest developing and implementing a nursing ethics education program that can help nurses enhance their moral sensitivity and effectively manage various ethical conflicts related to infection control performance in the context of emerging infectious diseases. Furthermore, we recommend developing guidelines to establish a safe environment within healthcare institutions and to create an effective infection prevention environment.

## Relevance to clinical practice

This study provides baseline data for developing policies and guidelines to maximize infection control performance among nurses in national and public hospitals regarding emerging infectious diseases. There is a need for programs aimed at enhancing moral sensitivity and developing guidelines for a safe environment.

### What does this paper contribute to the wider global clinical community?

- This study provides baseline data for developing policies and guidelines to maximize infection control performance among nurses in national and public hospitals regarding emerging infectious diseases.
- This study contributes to the development of evidence-based practice and scientific inquiry related to moral sensitivity and safe environments, which hold significant implications for enhancing infection control in the context of emerging infectious diseases.

## Data Availability

The datasets used and/or analyzed during the current study are available from the corresponding author on reasonable request.
